# Biological Activities of Seven Medicinal Plants Used in Chiapas, Mexico

**DOI:** 10.3390/plants11141790

**Published:** 2022-07-06

**Authors:** Liliana De La Cruz-Jiménez, Mario Alberto Hernández-Torres, Imelda N. Monroy-García, Catalina Rivas-Morales, María Julia Verde-Star, Vianey Gonzalez-Villasana, Ezequiel Viveros-Valdez

**Affiliations:** 1 Department of Chemistry, College of Biological Sciences, Universidad Autónoma de Nuevo León, Av. Pedro de Alba S/N, San Nicolás de los Garza 66450, Nuevo León, Mexico; lidelac.j@gmail.com (L.D.L.C.-J.); mario.hernandeztr@uanl.edu.mx (M.A.H.-T.); imelda.mg@mochis.tecnm.mx (I.N.M.-G.); catalina.rivasmr@uanl.edu.mx (C.R.-M.); maria.verdest@uanl.edu.mx (M.J.V.-S.); 2 Tecnológico Nacional de México, Instiuto Tecnológico de Los Mochis, Departamento de Ingeniería Química y Bioquímica, Juan de Dios Bátiz y 20 de Noviembre, Los Mochis 81259, Sinaloa, Mexico; 3 Department of Cellular Biology and Genetics, College of Biological Sciences, Universidad Autónoma de Nuevo León, Av. Pedro de Alba S/N, San Nicolás de los Garza 66450, Nuevo León, Mexico; vianey.gonzalezvl@uanl.edu.mx

**Keywords:** Chiapas, medicinal plants, antimicrobial, antioxidant, antihemolytic, anti-α-glucosidase activity, toxic effect

## Abstract

Seven medicinal plants from Chiapas, Mexico, used by Native Americans were analyzed, aiming to improve the understanding of their medicinal properties through the evaluation of various biological activities, i.e., bactericidal, antioxidant, α-glucosidase inhibition, and toxicity, to provide a scientific basis for the management of infectious and hyperglycemic diseases in the Mexican southeast. Plant extracts were obtained from *Cordia dodecandra*, *Gaultheria odorata*, *Heliotropium angiospermum*, *Justicia spicigera*, *Leucaena collinsii* spp. *collinsii*, *Tagetes nelsonii*, and *Talisia oliviformis* through maceration techniques using methanol and chloroform (1:1). Minimum Inhibitory Concentration (MIC) was employed to determine the antibacterial activity against *Staphylococcus aureus*, *Enterobacter faecalis*, *Escherichia coli*, *Enterobacter aerogenes*, *Enterobacter cloacae*, *Klebsiella pneumoniae*, and *Pseudomonas aeuroginosa.* The antiradical/antioxidant activity was determined by the 2,2-diphenyl-1-picrylhydrazyl (DPPH), 2,2’-azino-bis(3-ethylbenzothiazoline-6-sulfonic acid) (ABTS) assays and antihemolytic activity using the 2,2’-Azobis(2-amidinopropane) dihydrochloride radical (APPH). The anti-α-glucosidase activity was evaluated in vitro through the chromogenic PNPG assay. The toxicity was assessed using the brine shrimp lethality assay. The highest antimicrobial activity was displayed by *T. nelsonii*, mainly against *E. faecalis* and *P. aeuroginosa*. The extracts of *L. collinsii*, *J. spicigera*, and *T. nelsonii* possess antioxidant properties with EC_50_ < 50 μg/mL. *J. spicigera* and *T. nelsonii* extracts showed the highest antihemolytic activity with IC_50_ < 14 μg/mL. *T. nelsonii* exhibited a remarkable inhibitor effect on the α-glucosidase enzyme and the greatest toxic effect on *Artemia salina* with IC_50_ = 193 ± 20 μg/mL and LD_50_ = 14 ± 1 μg/mL, respectively. According to our results, *G. odorata*, *J. spicigera*, *T. nelsonii*, and *T. oliviformis* extracts contained active antimicrobial compounds. At the same time, *T. nelsonii* stands to be a possible source of effective antineoplastic and antihyperglycemic compounds.

## 1. Introduction

The use of medicinal plants has been a constant since ancient times, to such a degree that the World Health Organization (WHO) recognizes its important value. Nowadays, it is calculated that 80% of the world population makes use of these types of plants to treat illnesses and diseases [1]. Of the nearly 250,000–500,000 species of plants on earth, 20,000 have medicinal properties and function as a drug source [2], while 60–70% of the total worldwide number of medicinal plants are found in Latin America [3].

Mexico possesses 10% of the global flora, and its southeast region is endowed with a profound bio-cultural wealth, being the richest region of the country with more than 8000 species of vascular plants employed for medicinal purposes [4]. The major part of the Mexican tropical forest that possesses most of the flora and fauna species, including those under protection, is located in this area.

These tropical areas represent 11% of the total land of Mexico and point to the southeast as the most suitable area for the study of active compounds based on natural products [4,5]. 

In Mexico, between 15 and 20 million people use traditional medicine, highlighting its prolonged use in the southern region, where around 10 million indigenous people and 56 ethnic groups live, allowing an extended knowledge of the use of natural medicinal plants [6]. The southeast of Mexico is associated with unique vegetation, such as the Lacandon jungle, located in the Chiapas state, where many ethnic groups still reside, such as Choles, Chujes, Lacandones, Mames, Mochos, Jacaltecos, Tzeltales, Tojolabales, Tzotziles and Zoques [7]. Those ethnic groups keep their traditions and folklore along with their ancient herblore. 

On the other hand, infectious diseases are one of the main causes of morbidity and mortality in half of the developing countries. In Mexico, the National Institute of Statistics and Geography (INEGI) points out that around 20% of deaths in the Mexican south are due to infections [8]. However, the death rate increases when people have chronic diseases such as diabetes mellitus (DM). According to the information of herbal treatments, most of the diseases that are treated with medicinal plants in the country are those with symptoms related to bacterial and parasitic affections, including diarrhea, abdominal pain, dysentery, and parasites [9]. This epidemiologic profile is consistent with the reports of the Mexican Institute of Social Security (IMSS), relating the prevalence of infectious diseases to the indigenous areas of the country. Furthermore, the incidence of DM has increased due to globalization, modernization, and changes in human lifestyle. It is estimated that around 530 million people between 20 and 79 years of age are affected by diabetes in the world. In Mexico, more than 15% of the adult population suffers from this disease, which still has no cure [10,11]. 

One of the most common treatments applied in patients with DM is the use of postprandial carbohydrate absorption inhibitors. These drugs inhibit some enzymes, such as α-glucosidase but with undesirable side effects [12]. As a result, the potential of medicinal plants for this purpose has already been evaluated [13]. Furthermore, another prevention measure, along with a balanced diet and moderate exercise, is the supplementation of antioxidants in the diet, helping to reduce the presence of free radicals in the system, given that the close relationship between diabetes and oxidative stress is already well known [14]. Based on the above, the use of medicinal plants could contribute to the complementary treatment of DM, both by slowing the absorption of carbohydrates and providing antioxidants that protect the body from damage caused by oxidant agents.

During the last 20 years, the research in the field of natural products has re-emerged due to the discovery and development of new molecules with pharmaceutical interest based on the ethno-medical knowledge; more than 90% of the vegetal species have not been studied exhaustively yet [15]. For this reason, further studies in this area are needed. 

Therefore, based on epidemiological data and empirical knowledge, we studied medicinal plants that are commonly used by ethnic groups from southeast Mexico to probe their efficacy and corroborate their medicinal properties.

## 2. Results

### 2.1. Medicinal Plants

Seven medicinal plants used in Chiapas, Mexico, to treat gastrointestinal affections were collected. Table 1 provides specific information on each medicinal plant. 

### 2.2. Yield of Extraction and Phytochemical Screening

The obtained percent of yield extraction by a maceration technique and a mixture of CH_2_Cl_2_:MeOH (1:1) ranged between 1.17 for *T. oliviformis* and 5.92 for *G. odorata*. The phytochemical screening in the seven extracts showed the presence of secondary metabolites such as flavonoids, phenolic compounds, coumarins, alkaloids and steroids. Results for each extract are shown in Table 2. 

### 2.3. Antimicrobial Activity

Seven bacteria strains were used to determine the MIC. Extracts from *G. odorata*, *H. angiospermum*, *J. spicigera*, *T. nelsonii* and *T. oliviformis* demonstrated inhibitory effects against *E. faecalis* (Table 3). It was observed that *T. nelsonii* inhibits the growth of *E. cloacae*, *E. aerogenes* and *P. aeuroginosa* but also *J. spicigera*, and *T. oliviformis* inhibited this last strain. None of the analyzed extracts inhibit the growth of *K. neumoniae* or *E. coli*. The extracts from *C. dodecandra* and *L. collinsii* did not show any antibacterial activity by this assay.

### 2.4. Antioxidant, Antihemolytic, Anti-α-Glucosidase and Toxicity Activities

The antioxidant activity related to the DPPH and ABTS free radical scavenging effect of the extracts was compared with Trolox. Samples with an EC_50_ ≤ 50 μg/mL were considered with relevant antioxidant activity. *J. spicigera*, *L. collinsii* and *T. nelsonii* exhibited the greatest antioxidant effect. On the other hand, AAPH was used to form peroxyl radicals and induce oxidation in human erythrocytes. The anti-hemolytic effect was observed for extracts of *H. angioespermum*, *L. collinsii*, *J. spicigera* and *T. nelsonii*, where the last two had the greatest effect with mean inhibitory concentration values of 13.5 ± 4 and 9.2 ± 2 μg/mL, respectively. For the anti-α-glucosidase activity test, *T. nelsonii* extract exhibited a significant glucosidase enzymatic inhibition with an IC_50_ value of 193 ± 20 μg/mL with respect to acarbose (120 ± 20 μg/mL).

Regarding toxicity, the lethal dose (LD_50_) for an extract to be considered non-toxic is ≤1000 μg/mL, those between 1000 and 500 μg/mL are considered slightly toxic, between 500 and 100 μg/mL are moderately toxic, and between 100 and 10 μg/mL are considered highly toxic [30]. According to these criteria, *G. odorata*, *J. spicigera* and *L. collinsii* with LD_50_ of 780.9 ± 11 μg/mL, 841.2 ± 27 μg/mL and 820.5 ± 40 μg/mL, respectively, are slightly toxic; *C. dodecandra* (310.5 ± 15 μg/mL) and *H. angiospermum* (430.2 ± 20 μg/mL) are moderately toxic, and *T. nelsonii* with an LD_50_ of 14 μg/mL is highly toxic, with lower values than the positive control (K_2_Cr_2_O_7_), with LD_50_ of 18 μg/mL. All these biological activities are shown in Table 4.

## 3. Discussion

With the aim to contribute to the studies and knowledge of the Mexican ethnopharmacognosy, the present study evaluated some of the biological and chemical properties of seven native plants from the southeast of the country that are used traditionally for medicinal purposes.

For the extraction of bioactive compounds from medicinal plants, organic solvents are commonly used since they allow greater extraction of the compounds and easier handling of the extracts obtained due to the high point of evaporation of water, as well as the low extraction of compounds from moderate to low polarity [31]. Related to this, it has already been shown that the extraction mixture of CH_2_Cl_2_:MeOH (1:1) allows the extraction of a wide diversity of compounds, such as terpenes, flavonoids, iridoids, and saponins [32,33,34,35,36], with different biological activities (antibacterial, antifungal, cytotoxic, antioxidant, etc.). Moreover, a higher efficiency in the extraction of bioactive withanolides has been observed using this mixture of CH_2_Cl_2_:MeOH (1:1) solvents, instead of using dichloromethane, ethyl acetate, hexane, and methanol separately [37], allowing us to have in this way a more efficient investigation of the active extracts obtained from medicinal plants.

Regarding MIC results, it is considered that the strains with minimal inhibitory concentrations of 0.06 mg/mL or less are sensitive to the extracts, and those strains with a MIC between 0.12 and 2 mg/mL have a medium sensitivity, but those over 2 mg/mL are highly resistant [38]. According to these results and with the aforementioned information, *E. faecalis* (Gram-positive) is sensitive to the extracts of *H. angiospermum*, *J. spicigera* and *T. oliviformis*, highly resistant to the extracts of *C. dodecandra* and *L. collinsii,* and presents a medium sensitivity to the rest of the plants. The methanolic extract of *T. nelsonii* was previously reported with an MIC of 100 mg/mL against *P. mirabilis*, *S. aureus*, *P. aeruginosa* and *C. albican* [39]. The mixture of solvents used in the extraction of bioactives from *T. nelsonii* could have contributed to the fact that a greater biological effect was observed in our study. The bactericidal activity of *J. spicigera* and *T. oliviformis* was previously demonstrated against *E. coli* and *S. aureus* [40,41]. It is important to mention the effect of *T. nelsonii*, *J. spicigera* and *T. oliviformis* against *P. aeuroginosa*, considering that it is an important cause of infection, and there is an increasing antibiotic resistance for this strain [42]. Therefore, the extract of *J. spicigera* with an MIC of 0.5 mg/mL could open an opportunity to find interesting bioactive compounds.

*G. odorata* shows moderate activity against *S. aureus*, *E. faecalis*, *E. aerogenes* and *E. cloacae*, and the presence of ursolic acid could be related to this effect, as it was previously reported as the active compound of *G. odorata* against *S. aureus* [43]. *H. angiospermum* exhibited potent activity against *E. faecalis;* the effect of the ethanolic extract has already been demonstrated on *S. aureus*, *S. epidermidis*, *P. aeruginosa* and *K. pneumoniae* [44], pointing out that active compounds of this species are polar in nature, which could explain the differences to our data when the polarity decreased when mixing methanol and dichloromethane.

To demonstrate the antiradical/antioxidant effect, the hydrogen-donating potential of samples was evaluated by DPPH and ABTS radical-scavenging activity. The extracts from *J. spicigera*, *L. collinsii* and *T. nelsonii* could be considered to have powerful antioxidant activity, with *J. spicigera* being the one showing the best antioxidant results in both analysis; however, none of them had a similar effect to the positive control. However, the Trolox is a pure compound as opposed to extracts that have an unknown number. Antioxidant studies [45,46] from the genera *Tagetes* report an EC_50_ under 50 μg/mL and thus support the present results. Flavonoids and monoterpenoids found in these plant extracts could be responsible for their antioxidant activity, while previous reports indicate that quercetin and kaempferol are flavonoid compounds allowing this biological activity [47]. An analysis of *J. spicigera* in different states: fresh, dry and storage, shows that the phenolic compounds found in the plant provide the antioxidant activity, hence indicating that the best ABTS radical scavenging activity was traced in the fresh extract; according to this, we can suggest that we may be able to improve our results if fresh material is used in further studies [48].

Although free radicals used in in vitro assays are useful for demonstrating the antioxidant potential of a sample, it has recently been suggested that there is a need to show the effects in a more complex physiological system in more detail; thus, the powerful antioxidant effect shown by some species using the AAPH radical-induced hemolysis protection assay in human erythrocytes was confirmed since this system is considered as an ex vivo model [49]. The inhibitory activity of extracts against peroxyl radicals induced by an AAPH hemolytic agent was found as *Tagetes nelsonii* (IC_50_ 9.2 ± 2 μg/mL) > *J. spicigera* (IC_50_ 13.5 ± 42 μg/mL) > *L. collinsii* (IC_50_ 60.8 ± 172 μg/mL) where ascorbic acid, a standard antioxidant agent, displayed IC_50_ of 297 μg/mL ± 30. The antihemolytic activity of polar extracts of 30 plants was reported by Sangkitikomol in 2012. Plants such as Mexican marigold (*Tagetes erecta*), Guaje (*Leucaena leucocephala*), Guava (*Psidium guajava*), Rose (*Rosa domescena*), and Green tea (*Camellia sinensis*) showed enhanced protection, increasing the time required to reach 50% hemolysis above 210 min, compared to 120 min observed for the APPH agent. The protective role was attributed to the polyphenol content of the plant as these compounds interact with the erythrocyte membrane components through hydrogen bonds, which in turn prevents the oxidation of proteins and lipids of the membrane [49].

Antioxidants present in medicinal plants are considered important due to their numerous health benefits, as they contain a wide variety of molecules that scavenge free radicals, such as phenolic, alkaloids, and terpenoids [50]. On the other hand, free radicals may also be related to the development and complication of diabetes mellitus (DM) [51]. DM is a public health problem that is characterized by high blood glucose levels. The usual therapy for the treatment of diabetes is to postpone the absorption of glucose by inhibiting carbohydrate-hydrolyzing enzymes, such as α-glucosidase. Acarbose is the most extensively used α-glucosidase inhibitor but has gastrointestinal side effects [52]. As medicinal plants are potential sources of drugs, α-glucosidase inhibitors screened from plants have been studied intensively in recent years [53]. In this investigation, *Tagetes nelsonii* extract showed remarkable α-glycosidase inhibitory effects with EC_50_ = 193 ± 20 μg/mL, although lower activity than the hydrophilic extract of *Tagetes erecta* flower with EC_50_ = 60 μg/mL. This flower extract has rich phenolic acids (sinapic, ferulic, and p-coumaric acids) and flavonoids (kaempferol and myricetin), which are the most abundant compounds and the most enzymatic inhibitors possible [54].

One of the main objectives of research groups involved in the study of medicinal plants has been to develop and use cheap, reproducible, and easy-to-use assays. Within this group of bioassays, the lethality test on *Artemia* spp larvae stands out. [55,56]. This test is used to determine the general toxicity of samples (extracts, fractions and/or compounds). The correlation between the results reported on *Artemia salina* and mice has already been established [57]. All tested species except *T. nelsonii* showed moderate to low toxicity in the *Artemia* assay, and it is interesting to note that in our study *J. spicigera* (LD_50_ = 841.2 ± 27 μg/mL) did not exhibit severe toxicity, whereas Vega et al. [58] reported the cytotoxicity (ED_50_ < 20 μg/mL) of ethanolic extract against T47D and HeLa cancer cell lines, this difference may be due to the type of the analyzed extracts. Likewise, and based on the obtained results, *T. nelsonii* has the potential for the development of anticancer compounds due to the correlation between the *Artemia assay* with human solid tumor cell lines [51,59]. In relation to the above, 7-methoxycoumarin and 6,7-dimethoxycoumarin were isolated from the hexane leaf extract of *Tagetes lucida,* showing high cytotoxicity to *A. salina*, with LC_50_ values of 28 and 238 μg/mL [60]. 

On the other hand, some compounds present in the studied species (Table 1) have shown interesting biological activities. For instance, *C. dodecandra*, in which the cordiaquinone, menaquinone, quercetin, and rosmrinic acid have been studied for the design of antimicrobial and antineoplastic agents [61,62,63,64]. The amyrins and b-sitosterol reported in *H. angiospermum* have shown an important antioxidant and cytotoxic effect [65,66]. Likewise, preclinical studies of kaempferol have already been carried out in a wide range of pharmacological activities, including antioxidant, antimicrobial, anticancer, and antidiabetic properties [67], and this compound has also been reported in *J. spicigera*. Moreover, the monoterpenes dihydrotagetone, E-β-tagetone and ocimenones, present in the volatile fraction of *T. nelsonii*, have been reported to have an antifungal and antibacterial effect [68,69]. In our study, we observed that *T. nelsonii* has cytotoxic and antihyperglycemic compounds with high antioxidant capacity that merits further studies for the development of DM and cancer therapies. 

## 4. Materials and Methods

### 4.1. Plant Collections and Identification

Fresh plants were collected in Tuxtla Gutierrez, San Fernando and Zinacantan, State of Chiapas, Mexico. Samples were identified at the species level by the Botanic department at the Universidad Autónoma de Nuevo León. Specimens were deposited in the herbarium of the College of Biological Sciences, Universidad Autónoma de Nuevo León under the following voucher numbers; *Gaultheria odorata* (025884), *Cordida dodecandra* (025879), *Heliotropium angiospermum* (025880), *Talisia oliviformis* (025881), *Leucaena collinsii spp. Collinsii* (025882), *Tagetes nelsonii* (025883) and *Justicia spicigera* (25114). Table 1 provides the specific information about each plant.

### 4.2. Extraction

Different aerial parts were used for the extractions. For *G. odorata* and *H. angiospermum,* the leaves, stems and flowers were used; for *L. collinsii* and *T. oliviformis,* we used the seeds and leaves, respectively; for *C. dodecandra* only the bark and for *T. nelsonii* and *L. collinsii* exclusively stems and leaves were used. 

In each case, 30 g of the finely ground air-dried plants was extracted through the maceration technique with a mix of solvents dichloromethane: methanol (1:1). Afterward, three successive extractions by sonication for 20 min each were performed [37]. Extracts were filtered and concentrated using a rotary evaporator (Yamato Model RE2000). The extracts were weighed, and the percentage yields were calculated. The obtained extracts were stored at 4 °C until use.

#### Phytochemical Assay

The organic extracts were assessed for phytochemical screening using the methodology proposed by Harborne [70]. A colorimetric reaction, the appearance of solids or foam during the identification reactions, allows a semi-quantitative evaluation of the presence of secondary metabolites. All solvents and chemicals were purchased from Sigma-Aldrich Corp. (St. Louis, MO, USA).

### 4.3. Antimicrobial Activity

#### 4.3.1. Test Organisms

Clinical isolates of *Staphylococcus aureus*, *Enterococcus faecalis*, *Escherichia coli*, *Enterobacter aerogenes*, *Enterobacter cloacae*, *Klebsiella pneumoniae*, and *Pseudomonas aeuroginosa* were tested. A bacterial suspension was prepared using 24 h cultures of the above-mentioned microorganisms, and turbidity was adjusted to 1.0 McFarland standard, which corresponded to 1.5 × 10^8^ CFU/mL. 

#### 4.3.2. Antibacterial Activity Assay

The minimal inhibitory concentration (MIC) for each extract was determined in 96-well microplates by the broth microdilution method. The concentration of the extracts that caused complete inhibition of growth after 24 h of incubation was considered active [71]. Samples were dissolved in Mueller–Hinton medium (purchased from BD GmbH, Heidelberg/Germany) with 10% DMSO and 100 µL was added per well, making serial dilutions of each extract (0.016 to 2 mg/mL). Gentamycin was used as a positive control (16–0.063 × 10^−3^ mg/mL). Then, 5 µL of inoculum containing each microorganism was added to each well. All assays were performed in triplicate. 

### 4.4. Antioxidant/Antiradical Activities

#### 4.4.1. Antiradical Assays

DPPH (2,2-diphenyl-1-picrylhydrazyl) radical scavenging activity was determined by mixing 100 µL of extracts dissolved in methanol (from 100 to 6.25 μg/mL) and 100 µL of DPPH aqueous solution (2 mg/L) in a 96-well microplate. The decrease in absorbance at 517 nm was measured in a microplate reader; mean values were obtained from triplicate experiments [72]. To evaluate ABTS (2,2’-azino-di-(3-ethylbenzthiazoline sulfonic acid)) radical scavenging activity, the free radical was prepared by mixing 10 mM ABTS stock solution with 2.45 mM potassium persulfate and leaving the mixture for 16 h. Then, the ABTS solution was diluted with ethanol to absorb 0.7 ± 0.05 at 734 nm. The assay was conducted by adding 200 µL of ABTS solution and 20 µL of the extract samples; measurements were taken at 734 nm after 6 min in darkness [72]. Trolox (6-hydroxy-2,5,7,8-tetramethylchroman-2-carboxylic acid) was used as control. The percentage of inhibition was calculated by: % inhibition = [(Abs control − Abssample)/Abscontrol] × 100, where: Abscontrol: control absorbance, and Abssample: sample absorbance. Half effective concentration (EC_50_) was calculated from the dose–response curve obtained. The EC_50_ is the concentration (or dose) effective in producing 50% of the maximal response.

#### 4.4.2. Antihemolytic Assay

The AAPH (2-2-Azobis (2-amidinopropane dihydrochloride) was used to form peroxyl radicals and induce oxidation in red blood cells. The antihemolytic assay was performed according to the methodology proposed by Monroy-García et al. with slight modifications [73]. The approval to work with human blood was obtained from the Institutional Review Board (Universidad Autonoma de Nuevo Leon, Departamento de Quimica de la FCB: UANL-CA-180-2019) with previous informed consent. A total of 5 mL from a healthy male donor was collected by venipuncture in tubes with anticoagulant (EDTA). Then, the blood was centrifuged to separate erythrocytes from plasma at 1500 rpm for 12 min at 25 °C and washed three times with 10 mL of PBS (137 mM NaCl, 2.7 mM KCl, 10 mM Na_2_HPO_4_, and 1.8 mM KH_2_PO_4_) pH 7.4. After that, erythrocytes were suspended in 8 mL of PBS to achieve a density of 8 × 10^9^ cells/mL. For the inhibition of hemolysis assay, the erythrocytes suspension was mixed with the extracts dissolved in PBS at serial dilution concentrations of 8 to 500 μg/mL and 300 mM AAPH in PBS (1:1:1 *v*/*v*/*v*). The mixture was incubated at 37 °C for 3 h with agitation. Afterward, the mixture was diluted with eight volumes of PBS and centrifuged at 3000 rpm for 10 min. The absorbance (A) of the supernatant was recorded at 540 nm. Percent inhibition was calculated by the following equation: % Inhibition = [AAAPH − As]/[AAAPH × 100], where AAAPH is the absorbance of AAPH at 540 nm and As is the absorbance of the extracts at 540 nm. Half inhibitory concentration (IC_50_) was calculated from the dose–response curve obtained by plotting the percentage of hemolysis inhibition versus the extract concentration. 

### 4.5. Anti-α-Glucosidase Assay

The enzymatic inhibition activity was evaluated according to the chromogenic method described in the literature [73]. All enzymes and chemicals were purchased from Sigma-Aldrich Corp. (St. Louis, MO, USA). The glucosidase solution (0.8 U/mL on PBS pH 6.8) was incubated at 37 °C for 15 min. Then, a 1:1 mixture of extracts (dissolved on PBS pH 6.8) and α-glucosidase were incubated in 96-well plates at 37 °C for 15 min. Later, 50 µL of 625 mM *p*-nitrophenyl-α-D-glucopyranoside (PNPG) solution was added to each well and incubated for another 15 min. Finally, the reaction was stopped by adding 100 µL of 0.2 M Na_2_CO_3_. The absorbance was read at 405 nm. An acarbose solution was used as a control. The assay was performed in triplicate, and the data are expressed as mean ± standard deviation (SD). The half-maximum inhibitory concentration (IC_50_) values were determined through a probit regression. The percentage of inhibition was calculated using the following equation:

% inhibition = [(Abs control − Abssample)/Abscontrol] × 100

### 4.6. Toxic Activity Assay

The brine shrimp (*Artemia salina*) lethality bioassay was performed to detect the potential toxic activity of each plant, according to Meyer et al. [55]. *A. salina* cysts were obtained from Prodac-México, hatched and cultivated in the laboratory, and after 48-h incubation in a warm room, nauplii were exposed to three different sample concentration (1000, 500, 300, 100, 50 and 10 μg/mL) containing 10 organisms/5 mL and incubated at 25 °C for 24 h. Vials were then examined, the number of living and dead shrimp nauplii were counted, and the median lethal dose (LD_50_) was calculated. Sea water, DMSO (1%) and K_2_Cr_2_O_7_ were used as controls. 

### 4.7. Statistical Analysis

The values were expressed as mean ± SD (*n* = 3). The differences among means were performed using a one-way analysis of variance, followed by Tukey’s comparison of means. The statistical analysis was carried out using the SPSS (version 18) program. Statistical significance was considered at *p* < 0.05.

## 5. Conclusions

The present study makes several noteworthy contributions to Mexican ethnopharmacology since this is the first report of biological properties and preliminary phytoscreening for *C. dodecandra* and *L. collinsii*, endemic species from the tropical regions of the Mexican southeast. *T. oliviformis* showed the best bactericidal effect against Gram-positive and Gram-negative bacteria and did not show toxicity on *A. salina* nauplii. Furthermore, this is the first study to investigate the toxicity of *T. nelsonii* as a good candidate for possible cytotoxic and antihyperglycemic compounds with high antioxidant capacity, leaving a potential area for future research. Our findings provide valuable contributions and validate the common use of some of the plants used in southeast Mexico.

## Figures and Tables

**Table 1:** Scientific and common name, useful part, uses, and chemical compounds reported in the analyzed medicinal plants.

Scientific Name	Common Name	Collected Part	Traditional Application	Chemical Compounds
*Cordia dodecandra*	Cupapé	Cortex	Diarrhea [16,17]	Cordiaquinone, menaquinone, rosmarinic acid, allantoin, quercetin, syringin, salvianolic acid B [18,19]
*Gaultheria odorata*	Arrayán	Flora, folia and caulis	Fever, diarrhea, stomach pain [20,21]	Not Reported
*Heliotropium angiospermum*	Cola de alacrán	Flora, folia and caulis	Gastroenteritis, stomach pain, diarrhea [20,22]	1α-2α-epoxy-1β-hydroxymethyl-8α-pyrrolizidine, α-amyrin, β-amyrin y β-sitosterol, A-blumenol, B-blumenol, loliolide, putrescine, spermine, turneforcidine, platynecine [22,23]
*Justicia spicigera*	Muicle	Folia and caulis	Diarrhea, stomach pain, dysentery, anticancer properties [24,25]	Allantoin, Kaempferitrin, kaempferol, β-glucosyl-O-sitosterol, Cryptoxanthin [7,26]
*Leucaena collinsii*	Guash	Semina	Anthelmintic [16,27]	Not Reported
*Tagetes nelsonii*	Chilchahua	Folia and caulis	Diarrhea, parasites, abdominal pain [7]	Dihydrotagetone, E-β-tagetone, Z-β-tagetone, cis-tagetone, limonene, trans-β-ocimene, α-terpineol,9-epi-(*E*)-cariofilene, cis-muurola-4(14),5-diene, γ-gurjunene, γ-himachelene, γ-morfene [28,29]
*Talisia oliviformis*	Guaya	Folia	Abdominal pain, fever, diarrhea [7]	Not Reported

**Table 2:** Yield of extraction and phytochemical screening of the medicinal plant extracts.

Extract Plant	Yield (%)	Alk	Coum	Flav	Phen	RS	Ter
*C. dodecandra*	1.59	+	+	+	+	+	+
*G. odorata*	5.92	+	+	+	+	+	+
*H. angiospermum*	3.72	+	−	−	−	+	+
*J. spicigera*	1.92	++	−	+	+	+	+
*L. collinsii*	2.11	−	+	−	−	+	+
*T. nelsonii*	5.04	+	−	++	++	+	++
*T. oliviformis*	1.17	−	+	−	+	+	+

Alk: alkaloid, Coum: coumarins, Flav: flavonoids, Phen: phenolics, RS: reducing sugars, Ter: triterpenoids; (++): abundant, (+): present, (−)absent.

**Table 3:** Antimicrobial activity of the medicinal plant extracts.

	Minimum Inhibitory Concentration (mg/mL)						
Extract Plant	*S. aureus*	*E. faecalis*	*E. coli*	*P. aeuroginosa*	*E. aerogenes*	*K. neumoniae*	*E. cloacae*
*C. dodecandra*	>2	>2	>2	>2	>2	>2	>2
*G. odorata*	1	0.5	>2	>2	1	>2	1
*H. angiospermum*	1	0.06	>2	>2	>2	>2	>2
*J. spicigera*	0.5	0.06	>2	0.5	1	>2	>2
*L. collinsii*	>2	>2	>2	>2	>2	>2	>2
*T. nelsonii*	>2	0.13	>2	1	1	>2	0.5
*T. oliviformis*	1	0.06	>2	1	0.5	>2	1
* *Control*	0.001	0.001	0.002	0.001	0.001	0.004	0.001

* Gentamicin was used as positive control; *n* = 3.

**Table 4:** Antioxidant, antihemolytic, anti-α-glucosidase and toxicity activities of the medicinal plant extracts.

	Effective Doses or Median Lethal (μg/mL)				
Extract Plant	*DPPH*	*ABTS*	*AAPH*	*α-* *Glucosidase*	*Toxicity*
*C. dodecandra*	>100	>100	>500	>500	310.5 ± 15 ^c^
*G. odorata*	>100	>100	>500	>500	780.9 ± 11 ^a^
*H. angiospermum*	>100	75.6 ± 11 ^a^	351. 4 ± 65 ^a^	>500	430.2 ± 20 ^b^
*J. spicigera*	35.8 ± 3.5 ^b^	15.7 ± 0.8 ^d^	13.5 ± 4 ^c^	268 ± 15 ^b^	841.2 ± 27 ^a^
*L. collinsii*	42.5 ± 2.5 ^a,b^	39.7 ± 4.9 ^b^	60. 8 ± 17 ^b^	323 ± 50 ^a^	820.5 ± 40 ^a^
*T. nelsonii*	47.7 ± 3.3 ^a^	28.2 ± 1.7 ^c^	9.2 ± 2 ^c^	193 ± 20 ^c^	14 ± 1 ^d^
*T. oliviformis*	>100	>100	>500	>500	>1000
* *Control*	7.3 ± 2.5 ^c^	5.4 ± 0.2 ^e^	297 ± 30 ^a^	120 ± 20 ^d^	18 ± 3 ^d^

* K_2_Cr_2_O_7_ was used as positive control in toxicity assays; Trolox on DPPH and ABTS antioxidant assays; ascorbic acid and acarbose were used for AAPH-induced hemolysis assay and inhibition of α-Glucosidase assays, respectively; *n* = 3, *p* < 0.05.

## Data Availability

The original contributions generated for this study are included in the article; the data presented in this study are available on request from the corresponding author.
